# Corticosteroid receptor rebalancing alleviates critical illness-related corticosteroid insufficiency after traumatic brain injury by promoting paraventricular nuclear cell survival via Akt/CREB/BDNF signaling

**DOI:** 10.1186/s12974-020-02000-2

**Published:** 2020-10-25

**Authors:** Bin Zhang, Miao Bai, Xiaojian Xu, Mengshi Yang, Fei Niu, Fei Gao, Baiyun Liu

**Affiliations:** 1 Department of Neurosurgery, Beijing Tiantan Hospital, Capital Medical University, Beijing, China; 2 Department of Neurology, Tangdu Hospital, Fourth Military Medical University, Xi’an, China; 3 Beijing Neurosurgical Institute, Capital Medical University, Beijing, China; 4 Beijing Key Laboratory of Central Nervous System Injury and Department of Neurosurgery, Beijing Neurosurgical Institute and Beijing Tiantan Hospital, Capital Medical University, No.119 South Fourth Ring West Road, Fengtai District, Beijing, 100070 People’s Republic of China; 5 Nerve Injury and Repair Center of Beijing Institute for Brain Disorders, Beijing, China; 6 China National Clinical Research Center for Neurological Diseases, Beijing, China

**Keywords:** Glucocorticoids, Corticosteroid receptor balance, Critical illness-related corticosteroid insufficiency, Apoptosis, Traumatic brain injury

## Abstract

**Background:**

We previously found that high-dose methylprednisolone increased the incidence of critical illness-related corticosteroid insufficiency (CIRCI) and mortality in rats with traumatic brain injury (TBI), whereas low-dose hydrocortisone but not methylprednisolone exerted protective effects. However, the receptor-mediated mechanism remains unclear. This study investigated the receptor-mediated mechanism of the opposite effects of different glucocorticoids on the survival of paraventricular nucleus (PVN) cells and the incidence of CIRCI after TBI.

**Methods:**

Based on controlled cortical impact (CCI) and treatments, male SD rats (*n* = 300) were randomly divided into the sham, CCI, CCI + GCs (methylprednisolone 1 or 30 mg/kg/day; corticosterone 1 mg/kg/day), CCI + methylprednisolone+RU486 (RU486 50 mg/kg/day), and CCI + corticosterone+spironolactone (spironolactone 50 mg/kg/day) groups. Blood samples were collected 7 days before and after CCI. Brain tissues were collected on postinjury day 7 and processed for histology and western blot analysis.

**Results:**

We examined the incidence of CIRCI, mortality, apoptosis in the PVN, the receptor-mediated mechanism, and downstream signaling pathways on postinjury day 7. We found that methylprednisolone and corticosterone exerted opposite effects on the survival of PVN cells and the incidence of CIRCI by activating different receptors. High-dose methylprednisolone increased the nuclear glucocorticoid receptor (GR) level and subsequently increased cell loss in the PVN and the incidence of CIRCI. In contrast, low-dose corticosterone but not methylprednisolone played a protective role by upregulating mineralocorticoid receptor (MR) activation. The possible downstream receptor signaling mechanism involved the differential effects of GR and MR on the activity of the Akt/CREB/BDNF pathway.

**Conclusion:**

The excessive activation of GR by high-dose methylprednisolone exacerbated apoptosis in the PVN and increased CIRCI. In contrast, refilling of MR by corticosterone protects PVN neurons and reduces the incidence of CIRCI by promoting GR/MR rebalancing after TBI.

## Introduction

Critical illness-related corticosteroid insufficiency (CIRCI) is very common after acute respiratory distress syndrome, sepsis and septic shock, traumatic brain injury (TBI), burns, major surgery, and other acute severe illnesses that cause structural damage to the hypothalamus-pituitary-adrenal axis (HPA) [1]. CIRCI is characterized by inadequate cellular corticosteroid activity and excessive inflammatory activity that contributes to the severity of the patient’s critical illness and is associated with increased morbidity and mortality. However, the pathogenesis of TBI-related corticosteroid insufficiency in the acute phase is quite different from CIRCI in other severe diseases; the former seems to be more central in origin, whereas the latter is more contributed to adrenal damage [2–4]. Our previous study revealed that apoptosis of neurons in the paraventricular nucleus (PVN) of the hypothalamus was an important reason for TBI-associated CIRCI [5, 6]. Unfortunately, although corticosteroid insufficiency after TBI is associated with hemodynamic instability and increased mortality or unfavorable outcomes, assessment of the HPA axis and corticosteroid replacement therapy are rarely considered and remain controversial in current TBI management.

Since the 1970s, glucocorticoids (GCs), especially high-dose synthetic GCs become commonly administered after TBI, due to their powerful anti-inflammatory effect [7, 8], However, the outcomes of clinical studies remain controversial [9, 10], and one trial showed that high-dose methylprednisolone (MP) increased the mortality of severe TBI patients, and the mechanism remains unclear [11]. Although clinical studies have found that a low dose of hydrocortisone reduced mortality in patients with severe TBI, septic shock, and relative corticosteroid insufficiency, the current guidelines of the Brain Trauma Foundation still recommend against corticosteroid treatment in TBI patients unless new compounds or mechanisms are found [12]. Accordingly, our previous animal studies showed that high doses of MP and dexamethasone (DEX) increased neuronal apoptosis in the PVN and exacerbated TBI-related CIRCI which is a major cause of increased mortality [5]. Chen et al. reported that low-dose hydrocortisone but not MP had protective effects on neurons in the PVN and hippocampus after TBI [6], but the mechanism of the opposite effects of GCs on the survival of PVN neurons after TBI remains unclear.

Unlike synthetic GCs (MP/DEX), which mainly activate glucocorticoid receptor (GR), natural GCs (cortisol/corticosterone (CORT)) can activate both the GR and mineralocorticoid receptor (MR) in the brain, but have a 10-fold higher affinity for the MR than GR [13]. Previous studies have indicated that both the MR and GR are abundantly expressed in the hippocampus and PVN. The continuous activation of MRs is indispensable for the survival of neurons in the hippocampus, while excessive or prolonged activation of GRs inhibits neurogenesis, increases neuronal apoptosis, and subsequently causes cognitive, mood, and stress disorders [14, 15]. Our previous studies revealed that the use of the GR agonist DEX increased cell loss in the hippocampus and exacerbated spatial memory impairment by promoting the activation of GR and inhibiting the activation of MR. In contrast, low-dose CORT and fludrocortisone promoted the survival of hippocampal neurons by increasing the expression and activation of MR [16, 17]. However, the changes in GR/MR expression and activation in the PVN remain unclear, and whether the deleterious or protective effects of MP/DEX and CORT/hydrocortisone are mediated by the GR/MR balance and their downstream signal transduction mechanisms have not been investigated. Both the protein kinase B (PKB/Akt)/cAMP-responsive element-binding protein (CREB)/brain-derived neurotrophic factor (BDNF) pathway and B cell lymphoma-2 (Bcl-2) family proteins were reported to be crucial for the survival of neurons after TBI [18–21]. Additionally, experimental studies revealed that GCs and their receptors affected neuronal survival in the hippocampus by regulating the ratio of Bcl-2 and Bax [22]. However, the effects of GR and MR on the Akt/CREB/BDNF pathway and Bcl-2 family proteins in the PVN after TBI have not been reported and need further study.

In the present study, we hypothesized that the balance of MR/GR expression and activation is crucial for the survival of neurons in the PVN after TBI. High-dose MP increased cell loss in the PVN and exacerbated CIRCI by disturbing the GR/MR balance, whereas low-dose CORT promoted the survival of neurons in the PVN by activating MR, and the possible receptor-mediated downstream mechanisms involved changes in Bcl-2 family proteins and the Akt/CREB/BDNF pathway. We tested our hypothesis in rats that were treated with low and high doses of MP, CORT, and their receptor antagonists by using an experimental model of TBI.

## Materials and methods

### Animals

All experimental procedures were approved by the Capital Medical University Institutional Animal Care and Use Committee. Adult male Sprague-Dawley rats weighing 300–320 g (Beijing Vital River Experimental Animals Technology, Ltd., Beijing, China) were used in this study. The rats were housed individually with constant temperature (22 °C) and humidity (60%) with a standard 12-h light/dark cycle and were provided food and water ad libitum.

### Controlled cortical impact models

Controlled cortical impact (CCI) was used to induce a severe TBI model with a PCI3000 PinPoint Precision Cortical Impactor (Hatteras Instruments, Cary, NC, USA) as previously described [23]. Briefly, after the rat was anesthetized with 5% isoflurane inhalation and fixed on a small animal ventilator (RWD Life Science Co., Shenzhen, China) with continuous 2% isoflurane, a midline incision was made to expose the skull, and then a right unilateral craniotomy approximately 6 mm in diameter was conducted midway between bregma and lambda, leaving the underlying dura intact. The impact injury was performed with a circular impact tip (5 mm) (velocity, 4.0 m/s; compression time, 85 ms; and depth, 3.0 mm). Sham-operated rats received only a craniotomy without percussion. All procedures were performed on a thermal pad (37.0 ± 0.5 °C).

### Experimental groups and treatments

Based on CCI and treatments, the male rats (*n* = 360) were randomly divided into the following seven groups: (1) sham group and (2) CCI group rats received an equal volume of solvent once per day for 7 days; (3) low-dose MP (CCI + MPL), (4) high-dose MP (CCI + MPH), and (5) low-dose CORT group (CCI + CORT) rats were administered an intraperitoneal injection once per day for 7 days (the first injection was administered within 1 h after surgery, and the remaining injections were administered between 9 AM and 11 AM daily on days 2–7 after CCI) [low-dose MP (1 mg/kg for 5 days, 0.5 mg/kg for 1 day, and 0.25 mg/kg for 1 day); high-dose MP (30 mg/kg for 5 days, 15 mg/kg for 1 day, and 7.5 mg/kg for 1 day) [5, 6]; and CORT (1 mg/kg for 5 days, 0.5 mg/kg for 1 day, and 0.25 mg/kg for 1 day) (ab143597, Abcam, UK)]; and the (6) RU486 + MPH and (7) spironolactone (SPIRO) + CORT group rats were administered the MR antagonist spironolactone (SPIRO; 50 mg/kg) (ab141289, Abcam, UK) or the GR antagonist RU486 (50 mg/kg) (ab120356, Abcam, UK), respectively, twice a day (at 9 AM and 4 PM) for 1 day before CCI and 7 days after CCI [24]. All drug dosages were chosen based on pilot experiments from our laboratory and previous studies from the literature [5, 6, 24].

### Tissue preparation for histological examination and western blotting

The rats were sacrificed by decapitation on day 7 after injury. Brains (*n* = 6 per group) that were used for histological analysis were removed and fixed in 4% paraformaldehyde for 24 h. After fixation, the brains were embedded in paraffin, processed into 5-μm-thick coronal sections (at the level of the PVN, according to the Paxinos atlas of the rat brain for basic orientation, including the bregma regions from − 1.4 to − 2.2 mm as the PVN) [25], and subsequently affixed to poly-l-lysine-coated slides. Brains (*n* = 12 per group at each time point) that were used for western blot analysis were rapidly removed, and the hypothalamus surrounding the third ventricular area was removed with a 3-mm punch at 4 °C, frozen in liquid nitrogen and then stored at − 80 °C before further processing. Total (*n* = 6) and nuclear protein (*n* = 6) extraction was performed as previously described [17].

### Plasma corticosterone assay and CIRCI assessment

A total of 210 rats (*n* = 30 per group) were used to assess the incidence of CIRCI and mortality. As previously described, an electrical stimulation (ES) model was used to assess the function of the HPA axis. Our previous study found that the plasma CORT levels peaked at 30 min after ES and gradually recovered to the baseline levels by 24 h after TBI [5, 6]. Therefore, 30 min after ES was selected as the timepoint for testing the CORT level. The ratio of the post-ES peak value to the pre-ES baseline value of CORT was defined as the CORT increase index (CII), and a CII value less than 2.5 was regarded as acute CIRCI according to our previous study [5]. Blood samples (200 μl) were collected from the orbital sinus of each rat under inhaled anesthesia (on preinjury day 7 and postinjury day 7 before ES and at 30 min after ES). After centrifugation at 3000 rpm for 10 min, the light yellow transparent supernatant plasma was separated and stored at − 80 °C for further CORT measurement. A CORT ELISA kit (ab108821; Abcam, UK) was used to assess the plasma CORT level as previously described [5].

### TUNEL assay and corticotropin-releasing hormone immunofluorescence staining

TUNEL assay was utilized to observe apoptosis in the hippocampus using the In Situ Cell Death Detection Kit, POD (Roche, Germany) according to the manufacturer’s instructions. Briefly, tissue sections were dewaxed, rehydrated, and incubated with a proteinase K working solution (20 mg/ml in 10 mM Tris-HCl buffer, pH 7.5–8.0) for 30 min at room temperature. The sections were rinsed twice with 0.01 M phosphate-buffered saline (PBS, pH 7.4) and subsequently incubated with the TUNEL reaction mixture for 2 h at 37 °C. Brain sections were blocked with normal bovine serum for 30 min and then incubated with rabbit polyclonal anti-CRH (1:1000, ab8901, Abcam, UK) overnight at 4 °C. The sections were then washed with PBS and incubated with Alexa Fluor 647-conjugated donkey anti-rabbit IgG at room temperature for 2 h. Finally, the samples were counterstained with diamidino-2-phenylindole (DAPI) (Sigma-Aldrich, St. Louis, MO) for 10 min.

To evaluate the survival of active neurons in the PVN after TBI, corticotropin-releasing hormone (CRH)- and TUNEL-positive cells in bilateral PVN areas were counted in three sections (at 50-μm intervals) obtained from bregma − 1.8 to − 2.2 mm that included the magnocellular and parvocellular regions. Digital images of the whole brain sections were obtained by a MIDI FL (3D Histech, Hungary) system. Then, a digital image analysis (DIA) was performed using a 3DHISTECH HQ software (3D Histech, Hungary). The data are presented as the total number of CRH- and TUNEL-positive cells in three sections. All assessments were performed by observers who were blinded to the experimental procedure.

### Double immunofluorescence staining

Brain slices were double-stained for immunohistochemical evaluation using fate-specific antibodies including CRH/BDNF and GR/MR. Briefly, the brain sections were incubated in 3% hydrogen peroxide for 0.5 h and then blocked with normal bovine serum for 0.5 h. The brain sections were washed three times and incubated with rabbit polyclonal anti-CRH (1:1000, ab8901, Abcam, UK), mouse monoclonal anti-BDNF (1:500, ab108319, Abcam, UK), rabbit polyclonal anti-GR (1/200 dilution; ab3578, Abcam, UK), and mouse monoclonal anti-MR (1/200 dilution; ab2774, Abcam, UK) overnight at 4 °C. The slices were then washed with PBS and incubated with Alexa Fluor 647-conjugated donkey anti-rabbit IgG and Alexa Fluor 488-conjugated goat anti-mouse IgG at room temperature for 2 h. Finally, the samples were counterstained with DAPI (Sigma-Aldrich, St. Louis, MO) for 10 min. Digital images of the whole brain sections were obtained by a MIDI FL (3D Histech, Hungary) system.

### Western blot analysis

Total (*n* = 6 per group) and nuclear protein (n = 6 per group) extraction were performed as previously described [16, 17]. Equal amounts of proteins (30 μg) were separated by SDS-PAGE and then transferred onto polyvinylidene fluoride membranes. The membranes were blocked with blocking buffer (5% milk or BSA) at room temperature for 2 h and subsequently incubated with primary antibodies at 4 °C overnight. The membranes were washed three times and incubated with secondary antibodies for 1 h at room temperature. The blots were developed using chemiluminescence (Bio Spectrum 500 Imaging System; UVP Co., Upland, CA, USA). The band densities were quantified using the Image J software (National Institutes of Health, Bethesda, MD, USA). The percent expression compared with that of the sham controls was calculated for each sample. The primary antibodies used were as follows: rabbit polyclonal anti-GR (1/200 dilution; ab3578, Abcam, UK), mouse anti-MR (1:400, ab2774, Abcam, UK), rabbit polyclonal anti-CRH (1:1000, ab8901, Abcam, UK), rabbit anti-Bcl-2 (1:1000; ab59348, Abcam, UK), rabbit anti-Bax (1:5000; ab32503, Abcam, UK), rabbit anti-Bad (1 μg/ml, ab90435, Abcam, UK), rabbit anti-P-Bad (S136, 1:1000, ab28824, Abcam, UK), mouse monoclonal anti-BDNF (1:5000, ab108319, Abcam, UK), rabbit anti-CREB (1:1000; ab31387, Abcam, UK), rabbit anti-P-CREB (ser133, 1:5000; ab32096, Abcam, UK), rabbit anti-Akt (1:500; ab8805, Abcam, UK), rabbit anti-P-Akt (ser 473, 1:5000; ab81283, Abcam, UK), rabbit anti-Actin (1:5000; ab179467, Abcam, UK), and anti-Histone H3 (1:400; Millipore Co.). The percent expression compared to that of the sham controls was calculated for each sample.

### Statistical analysis

All data are presented as the means ± standard deviation (SD). The data were analyzed using SPSS 22.0 (IBM Corporation, USA). Data on plasma CORT, CII, neuron counts, and western blotting were analyzed by one-way ANOVA. Kaplan-Meier survival analysis with the log-rank significance test was used to measure the mortality rates among rats with different treatments and the relationship between the incidence of acute CIRCI and mortality. In addition, the incidence of acute CIRCI was analyzed by chi-square tests. A *P* value < 0.05 was considered statistically significant.

## Results

### The opposite effects of CORT and MP on plasma CORT, CII, incidence of CIRCI, and mortality in rats after TBI

**Fig. 1:**
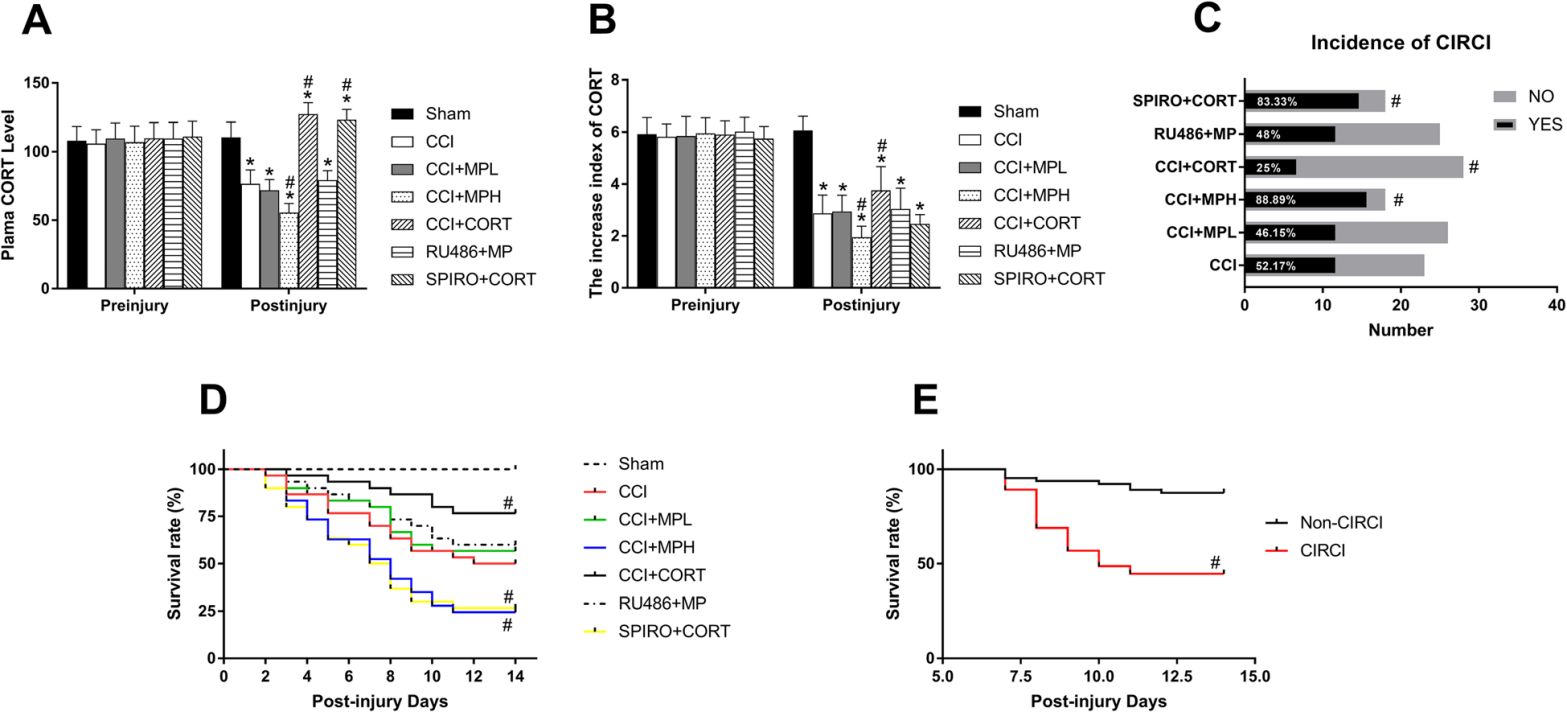
Effects of MP and CORT treatment on plasma CORT levels, CII, incidence of CIRCI and survival rate of rats after TBI. **a** Plasma CORT levels in the experimental groups before and after TBI. **b** Quantification of CIRCI increase index before and after TBI. **c** Incidence of CIRCI (*n* = 30 per group before injury; on postinjury day 7, *n* = 23 from the CCI group, *n* = 26 from the CCI + MPL group, *n* = 18 from the CCI + MPH group, *n* = 28 from the CCI + CORT group, *n* = 25 from the CCI + RU486 + MP group, *n* = 18 from the CCI + SPIRO+CORT group). **d** The survival of rats in the different groups after TBI (*n* = 30 per group). **e** The survival rate of rats with and without CIRCI (*n* = 74 from the CIRCI group and *n* = 64 from the non-CIRCI group). ^*^P < 0.05 versus the sham control group; ^#^*P* < 0.05 versus the TBI control group

In the present study, to evaluate the stress function before and after TBI, we tested the plasma CORT level, CII, and CIRCI. There was no significant difference in the plasma CORT levels and CII among the experimental groups before injury [one-way ANOVA, *F* (6, 203) = 0.814, *P* > 0.05 for plasma CORT; one-way ANOVA, *F* (6, 203) = 0.739, *P* > 0.05 for CII]. On postinjury day 7, the plasma CORT level and CII were significantly (*P* < 0.05) reduced in the CCI group. The high-dose MP treatment further inhibited endogenous CORT secretion; accordingly, the plasma CORT level and CII in the MPH group were significantly (*P* < 0.05) lower than those in the CCI group. In contrast, a low dose of CORT increased the plasma CORT to a level that was slightly above that in the sham group and increased the CII [one-way ANOVA, *F* (6, 161) = 236.37, *P* < 0.01 for CORT level; one-way ANOVA, *F* (6, 161) = 105.8, *P* < 0.01 for CII] (Fig. 1a–b). The inhibitory effect of high-dose MP on endogenous CORT secretion was counteracted by RU486. Accordingly, the chi-square test showed that the incidence of CIRCI in the MPH group was higher than that in the CCI group on postinjury day 7, whereas the CORT replacement significantly reduced the incidence of CIRCI compared with that in the TBI control group (chi-square = 23.536, df = 5, *P* < 0.01) (Fig. 1c). Pretreatment with RU486 and SPIRO counteracted the effects of MP and CORT, which supported that their effects were mediated by GR and MR.

In addition, CORT and MP exhibited opposite effects on the mortality of rats after TBI, and the MP-induced deleterious effects were dose-dependent. High-dose but not low-dose MP significantly increased (*P* < 0.05) the mortality rate of rats compared with the TBI control group. In contrast, low-dose CORT but not MP significantly reduced (*P* < 0.05) mortality (chi-square = 55.758, df = 6, *P* < 0.01) (Fig. 1d). Moreover, the incidence of CIRCI was positively correlated with mortality (chi-square = 27.663, *P* < 0.01) (Fig. 1e). The deleterious and protective effects of MP and CORT were counteracted by the GR antagonist RU486 and the MR antagonist SPIRO, respectively. Altogether, these results suggest that CIRCI is among the important factors contributing to the increased mortality after severe TBI and that the opposite effects of MP and CORT on CIRCI were mediated by the GR and MR, respectively. In addition, the effect of MP on CIRCI was dose-dependent; high-dose but not low-dose MP had obvious inhibitory effects on the activity of the HPA axis.

### The opposite effects of CORT and MP on apoptosis, the number of CRH-positive cells, and CRH expression in the PVN after TBI

Apoptosis is the main form of secondary cell loss contributing to approximately two thirds of cell death after TBI. In our previous study, we evaluated neuronal apoptosis in the PVN and found that apoptotic cells appeared on postinjury day 3 and peaked on day 7. The process of apoptosis is transient, and the number of apoptotic cells does not completely reflect the overall cell viability in the PVN after TBI. CRH is mainly secreted by the PVN neurons; thus, the number of CRH-positive cells in the PVN is an important parameter to evaluate the function of the PVN. Therefore, in the present study, we assessed PVN function using apoptosis, CRH-positive cells, and the expression of CRH on postinjury day 7.

**Fig. 2:**
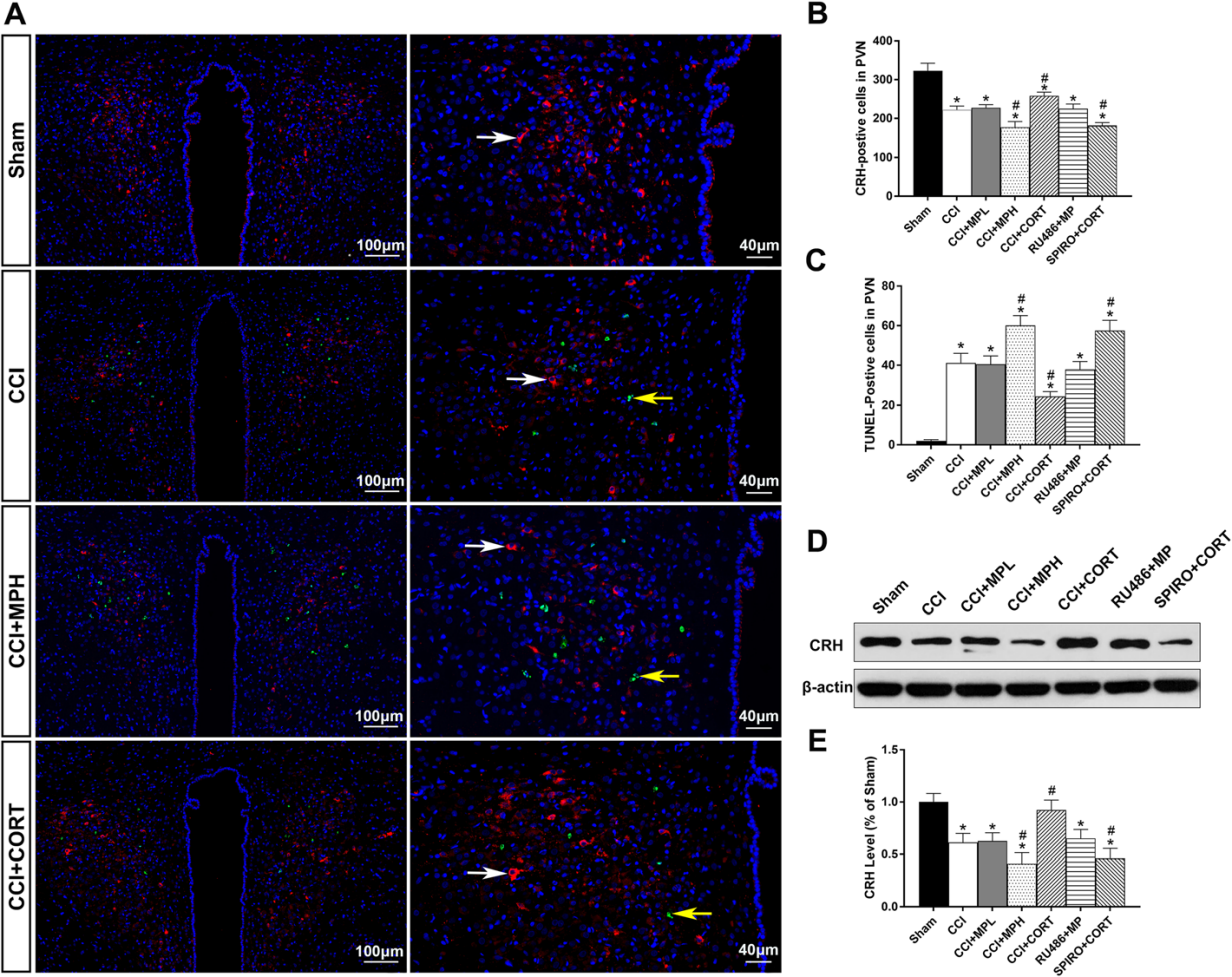
Effects of MP and CORT on the expression of CRH and the number of CRH- and TUNEL-positive cells in the ipsilateral hippocampus after TBI. **a** Representative images of CRH- (red, white arrow) and TUNEL-(green, yellow arrow) positive cells in the PVN. **b** Quantification of CRH-positive cells in the PVN. **c** Quantification of TUNEL-positive cells in the PVN. **d** Representative western blot images showing CRH expression. **e** Quantification of CRH expression. The relative band density was measured with ImageJ (1.49 V) and normalized to that of β-actin, and the percent expression compared to that of the sham controls was calculated for each sample. ^*^P < 0.05 compared to the sham group; ^#^*P* < 0.05 compared to the CCI control group. The data are presented as the means ± SD of 6 animals per group

The results showed that the number of CRH-positive neurons was significantly reduced after TBI, whereas the number of apoptotic cells in the PVN was significantly increased (*P* < 0.05) in the CCI group compared to that of the sham group (*P* < 0.05). High-dose MP treatment significantly reduced the number of CRH-positive neurons and increased the number of apoptotic cells compared to CCI group (*P* < 0.05). However, low-dose MP had no obvious effects on apoptosis and the number of CRH-positive neurons. In contrast, low-dose CORT replacement promoted the survival of CRH-positive neurons and significantly inhibited apoptosis compared with CCI control (*P* < 0.05) [one-way ANOVA, *F* (6, 35) = 101.48, *P* < 0.01 for CRH-positive cells; *F* (6, 35) = 143.98, *P* < 0.05 for apoptosis] (Fig. 2a–c).

Accordingly, western blotting showed that the expression of CRH in the PVN was significantly reduced on postinjury day 7 (Fig. 2d–e). A high dose of MP further inhibited the expression of CRH compared with CCI, while low-dose CORT replacement significantly increased the CRH level (*P* < 0.05) [one-way ANOVA, *F* (6, 35) = 35.58, *P* < 0.01]. Pretreatment with RU486 and SPIRO counteracted these effects of MP and CORT, respectively, on cell survival and CRH expression, which supported that these effects were GR- and MR-mediated. These results suggested that the secondary injury to hypothalamus is very common and represents an important factor leading to CIRCI after severe TBI. High-dose MP and low-dose CORT exhibited opposite effects by activating different receptors.

### The expression and activation of MR and GR were differentially regulated by CORT and MP

**Fig. 3:**
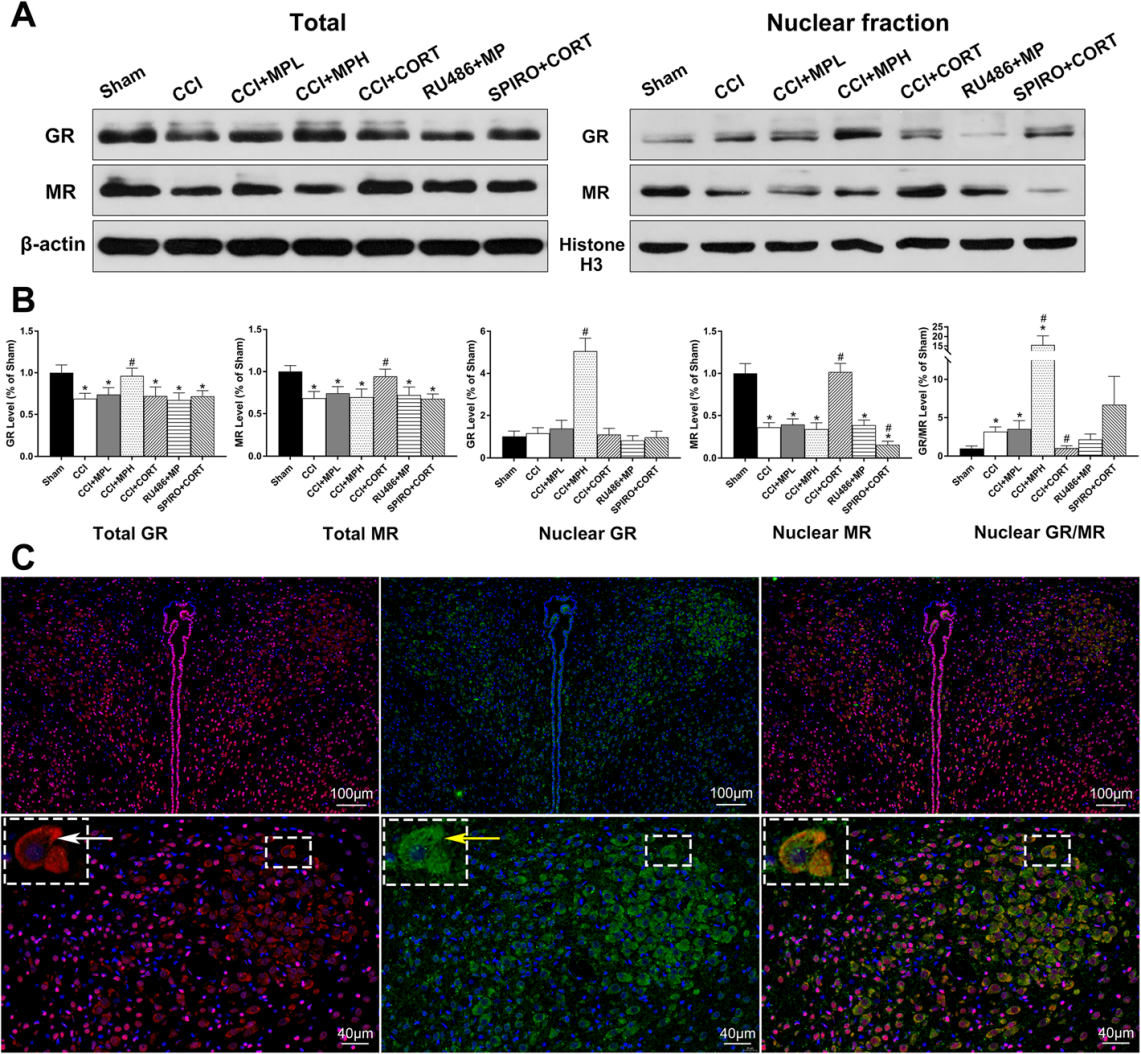
MP and CORT differentially affected neuronal survival in the PVN by regulating the expression and activation of MR and GR after TBI. **a** Representative western blot images showing total/nuclear MR and GR expression. **b** Quantification of total/nuclear MR and GR expression. The relative band density was measured with ImageJ (1.49 V) and normalized to that of β-actin/histone H3, and the percent expression compared to that of sham controls was calculated for each sample. **c** Representative images of GR (red, white arrow) and MR (green, yellow arrow) immunofluorescence in the PVN. ^*^P < 0.05 versus the sham control group; ^#^*P* < 0.05 versus the TBI control group. The data are presented as the means ± SD of 6 animals per group

MR and GR translocate from the cytoplasm to the nucleus when activated by their ligands. Therefore, the nuclear translocation of MR and GR is an important parameter for assessing their activation levels. We tested the total and nuclear GR and MR in the present study. We found that GR and MR in the PVN were significantly (*P* < 0.05) reduced in the CCI group compared to the sham group on postinjury day 7. However, the activation ratio of GR/MR was significantly (*P* < 0.05) increased. The CORT treatment significantly increased MR expression, while high-dose but not low-dose MP significantly increased GR expression (*P* < 0.05) [one-way ANOVA, *F* (6, 35) = 14.82, *P* < 0.01 for GR; *F* (6, 35) = 15.88, *P* < 0.01 for MR]. Similarly, the nuclear translocation of the MR in the PVN was significantly (*P* < 0.05) reduced in the CCI group compared to that in the sham group. Low-dose CORT replacement significantly (*P* < 0.05) increased nuclear MR and restored the ratio of GR/MR to the normal level compared with sham group (*P* > 0.05). In contrast, the high-dose but not low-dose MP treatment significantly (*P* < 0.05) increased nuclear GR and the ratio of GR/MR. The RU486 and SPIRO pretreatment inhibited the activation of GR and MR induced by CORT and MP, respectively (Fig. 3a, b) [one-way ANOVA, *F* (6, 35) = 109.10, *P* < 0.01 for nuclear GR; *F* (6, 35) = 114.99, *P* < 0.01 for nuclear MR; *F* (6, 35) = 28.95, *P* < 0.01 for the ratio of GR/MR]. These results suggested that the opposite effects of MP and CORT on PVN cell survival and the incidence of CIRCI were mediated by GR and MR respectively. Inadequate activation of MR occurred after TBI, and the ratio of GR/MR was reversed by CCI and high-dose MP treatment. In contrast, low-dose CORT restored the GR/MR balancing by upregulating the activation of the MR.

Figure 3 c is the representative image of immunofluorescence staining showing the distribution of the GR (red, white arrow) and MR (green, yellow arrow) in the PVN. Both GR and MR were abundantly coexpressed in PVN neurons.

### The effects of CORT and MP on the expression and activation of Bcl-2 family proteins

**Fig. 4:**
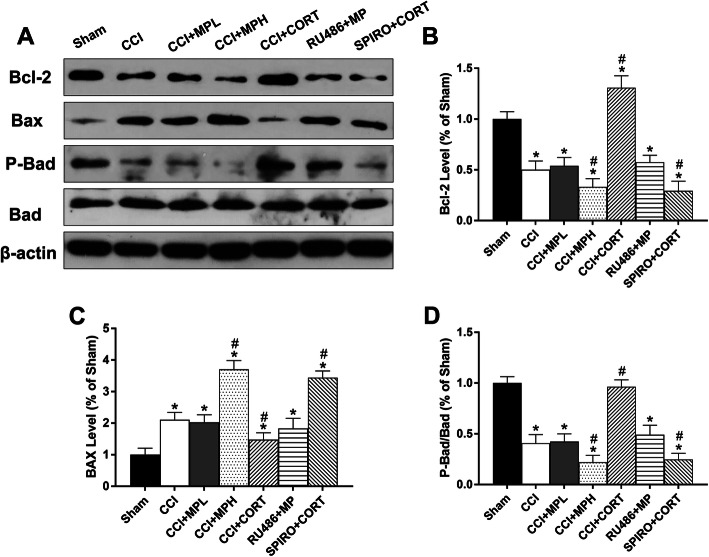
The opposite effects of MP and CORT on the expression and activation of apoptosis-modulating proteins. **a** Representative western blot images showing Bcl-2, Bax, P-Bad, and Bad expression. **b**–**d** Quantification of Bcl-2, Bax, and P-Bad/Bad levels. The relative band density was measured with ImageJ (1.49 V) and normalized to that of β-actin, and the percent expression compared to that of sham controls was calculated for each sample. ^*^P < 0.05 compared to the sham group; and ^#^*P* < 0.05 compared to the CCI control group. The data are presented as the means ± SD of 6 animals per group

BCL-2 family proteins play important roles in apoptosis, and the appropriate expression of these proteins is crucial for the survival of neurons after TBI. We tested the levels of anti-apoptotic (Bcl-2) and pro-apoptotic (Bax and Bad) proteins. Figure 4 showed the expression and activation of Bcl-2 family proteins after TBI and the GCs treatment. The Bcl-2 and P-Bad/Bad levels were significantly (*P* < 0.05) reduced in the PVN at 7 days after TBI, whereas Bax expression was significantly (*P* < 0.05) increased. CORT treatment significantly (*P* < 0.05) increased the expression of Bcl-2 and P-Bad/Bad but reduced Bax compared with the CCI group. In contrast, high-dose but not low-dose MP treatment significantly (*P* < 0.05) reduced the levels of Bcl-2 and P-Bad/Bad but increased Bax compared with the CCI group [one-way ANOVA, *F* (6, 35) = 107.86, *P* < 0.01 for Bcl-2; *F* (6, 35) = 96.17, *P* < 0.01 for Bax; *F* (6, 35) = 136.94, *P* < 0.01 for P-Bad/Bad]. The effects of MP and CORT on the BCL-2 family proteins were counteracted by RU486 and SPIRO, respectively, which demonstrated that these effects were receptor mediated. These results suggested that Bcl-2 family proteins played an important role in the regulation of cell apoptosis in the PVN after TBI. The differential effects of MP/CORT on the expression and activation of Bcl-2 family proteins might be an important factor contributing to their opposite effects on apoptosis.

### The Akt/CREB/BDNF pathway was involved in the opposite effects of the MR and GR on PVN neurons

**Fig. 5:**
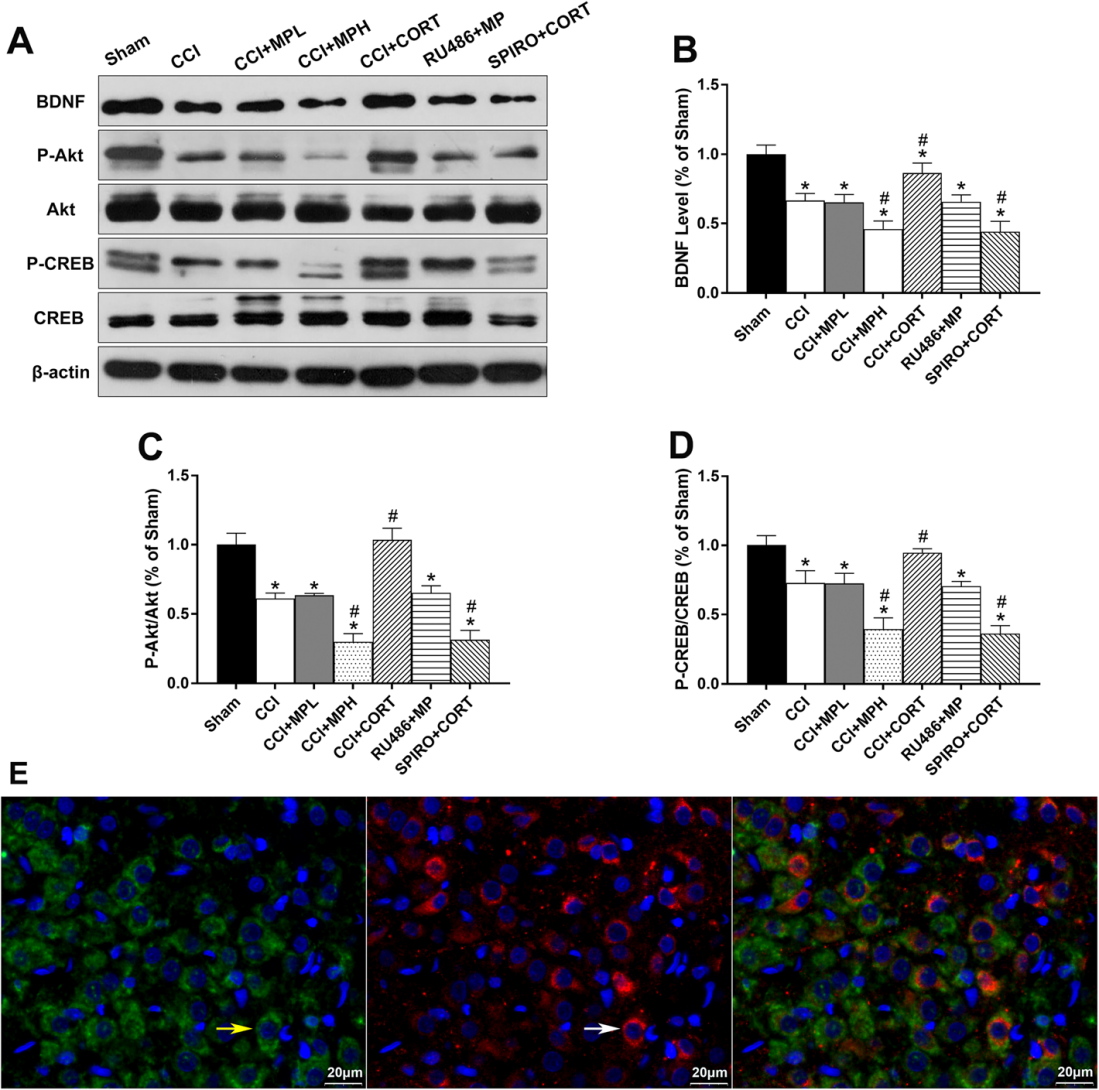
Akt/CREB/BDNF might be the possible downstream signaling pathway of GR- and MR-mediated effects on apoptosis in the PVN after TBI. **a** Representative western blot images showing BDNF, p-Akt, Akt, P-CREB, and CREB expression. **b**–**d** Quantification of BDNF, p-Akt/Akt, and p-CREB/CREB levels. The relative band density was measured with Image J (1.49 V) and normalized to that of β-actin, and the percent expression compared to that of sham controls was calculated for each sample. **e** Representative images of BDNF (green, white arrow) and CRH (red, yellow arrow) immunofluorescence in the PVN. ^*^P < 0.05 compared to the sham group, and ^#^*P* < 0.05 compared to the CCI control group. The data are presented as the means ± SD of 6 animals per group

Numerous intracellular signaling pathways participate in regulating neuronal apoptosis after TBI. Akt/CREB/BDNF is an important signaling pathway that is involved in secondary brain injury and plays a crucial role in regulating neuronal survival and apoptosis by affecting the activation and expression of Bcl-2 family proteins. Therefore, we examined the activity of the Akt/CREB/BDNF pathway after CCI and the GCs treatment. One-way ANOVA [*F* (6, 35) = 139.54, *P* < 0.01 for P-Akt/Akt; *F* (6, 35) = 71.52, *P* < 0.01 for P-CREB/CREB] showed that the ratios of P-Akt/Akt and P-CREB/CREB in the PVN were significantly reduced on postinjury day 7. High-dose but not low-dose MP treatment significantly (*P* < 0.05) reduced the ratios of P-Akt/Akt and P-CREB/CREB compared with CCI. In contrast, CORT replacement significantly increased the ratios of P-Akt/Akt and P-CREB/CREB. Accordingly, BDNF levels were significantly (*P* < 0.05) reduced at 7 days after TBI. Compared with the level in the CCI group, the BDNF expression level in the CCI + CORT group was significantly (*P* < 0.05) increased. In contrast, high-dose but not low-dose MP treatment significantly (*P* < 0.05) reduced BDNF expression [one-way ANOVA, *F* (6, 35) = 62.43] (Fig. 5a–d). The effects of MP and CORT on the expression of BDNF and the activation of Akt/CREB were counteracted by RU486 and SPIRO, respectively, which demonstrated that these effects were mediated by the two receptors. These results suggested that the activity of the Akt/CREB/BDNF pathway, which is a crucial upstream signaling pathway participating in the regulation of Bcl-2 family proteins and apoptosis process, was differentially regulated by MP and CORT via activating the GR and MR.

Figure 5e is the representative image of double immunofluorescence staining for CRH (red, white arrow) and BDNF (green, yellow arrow) in the PVN.

## Discussion

In the present study, we selected different doses of MP and low-dose CORT and examined their differential effects on the incidence of CIRCI, mortality, the survival and activity of PVN neurons, the receptor-mediated mechanism, and the possible downstream signaling pathways. We found that MP and CORT exhibited opposite effects on PVN cell survival and the incidence of CIRCI, and the deleterious effects of MP were dose dependent. High-dose MP upregulated the expression and activation of the GR but not MR, and subsequently increased the number of apoptotic cells, reduced the number of CRH-positive cells in the PVN, and increased the incidence of CIRCI and mortality. In contrast, low-dose CORT exerted protective effects by upregulating the expression and activation of MR. The possible downstream receptor signaling mechanisms involved the different effects of GR and MR on the Akt/CREB/BDNF pathway, which plays important roles in neuronal apoptosis and survival by regulating the expression and activation of apoptosis-related proteins.

The incidence of CIRCI is reported to be very high, varying from 10% in patients with other critical illnesses to as high as 60% in septic shock patients [1, 26]. In contrast, corticosteroid insufficiency is much higher in the acute phase after TBI than that of other conditions, occurring at rates from 54 to 78% [27, 28]. Compared with other forms of hypopituitarism, HPA axis dysfunction should be the greatest concern during the first 2 weeks after TBI because severe hyponatremia, hypotension, or hypoxia induced by corticosteroid insufficiency are associated with increased mortality. Adrenal necrosis and hemorrhage are reported to be the leading causes of CIRCI in severe acute illnesses other than TBI [1, 29], even though hypothalamus or pituitary injury, including ischemia, hemorrhage, and apoptosis, were also found in some patients with CIRCI after septic shock [30]. In contrast, most TBI-induced CIRCI is of central origin [3]. Pituitary hemorrhage/necrosis was found in more than 50% of patients with severe TBI by postmortem examination. In addition, up to 42% of patients have been reported to suffer hypothalamic injury [3, 4, 31, 32]. Our previous experimental studies also found that neuronal apoptosis in the PVN, which peaked at 7 days, was an important cause of CIRCI [5]. High doses of DEX or MP exacerbate apoptosis in the PVN and subsequently increase the incidence of CIRCI.

Similarly, the present study found that the high-dose MP treatment affected the activity of the hypothalamus by increasing neuronal apoptosis and downregulating CRH expression. CORT, the primary endogenous GC in rats, exhibited neuroprotective effects on PVN neurons and increased the CRH level. Accordingly, the incidence of CIRCI was increased after the high-dose MP treatment on day 7 after TBI, while the low-dose CORT replacement not only restored the plasma CORT level but also reduced the incidence of CIRCI and mortality. In addition, TBI-induced CIRCI had a strong positive correlation with mortality, suggesting that CIRCI was one of the major reasons for the increased mortality caused by high-dose MP. Both the MR and GR play crucial roles in regulating the function of the limbic-hypothalamic-pituitary-adrenal (HPA) circuit and stress activity. High endogenous and exogenous plasma GC levels prevent these stress reactions from overshooting by activating GR, and inhibits the secretion of endogenous GCs, causing suppression towards baseline conditions again [33]. However, both positive and negative feedbacks of MR to HPA axis have been reported [34, 35]. Our results showed that GR-specific antagonist RU486 counteracted the inhibitory effect of high-dose MP on the HPA axis and increased the plasma CORT, while the effects of SPIRO on endogenous CORT secretion and the plasma CORT level were not obvious because of the exogenous CORT treatment, which restored the plasma CORT to a high level.

Interestingly, we previously revealed that high-dose synthetic GCs (DEX or MP) increased the incidence of CIRCI after TBI by promoting apoptosis in neurons in the PVN, subsequently increasing mortality after TBI, while low-dose endogenous GCs (HP or CORT) but not MP exhibited the opposite effects [5, 6], but the mechanism remained unclear. By using immunohistochemistry, receptor autoradiography, and in situ hybridization, numerous studies have consistently found that MR and GR are highly coexpressed in the hippocampus (CA1 and dentate gyrus (DG)). However, the coexpression of MR and GR in the PVN is still controversial. In 1968, McEwen et al. first showed that a low dose of ^3^H-corticosterone, which primarily binds with MR, was abundantly retained in the hippocampus but not PVN in adrenalectomized rats [36]. Subsequent studies demonstrated that MR was also highly expressed in both the magnocellular and parvocellular regions of the PVN [37]. GR and MR can interact with each other or even bind to form heterodimers and may act in complementary or opposite ways depending on cell type or microenvironment [15, 38]. In animal models of chronic stress and depression, the balance of GR/MR expression and activation is found to be the prerequisite condition for maintaining the normal function and structure of hippocampal neurons. Over or prolonged activation of GR or insufficient activation and expression of MR inhibit neurogenesis and promote apoptosis and dendrite atrophy in hippocampal neurons, resulting in cognitive, mood, and neuroendocrine disorders [39, 40]. Our recent studies found that short-term use of high-dose DEX also disturbed the balance of GR/MR and subsequently increased the apoptosis of hippocampal neurons in rats after TBI, while low-dose CORT reduced neuronal apoptosis by activating the MR [16, 17]. However, the coexpression of and changes in GR/MR action in the PVN and their effects on the survival of neurons after TBI remains unclear.

In the present study, both the western blot and immunofluorescence revealed the coexpression of the MR and GR in the PVN. MR and GR expression and activation were reduced in the PVN after TBI. However, the ratio of GR/MR was significantly increased. The use of high-dose MP upregulated the expression and activation of the GR, further increased the activation ratio of GR/MR, and subsequently increased cell loss in the PVN. In contrast, a low dose of CORT increased the survival of neurons in the PVN by increasing MR activation and restoring the GR/MR balancing after TBI. Interestingly, although low-dose MP also increased the nuclear translocation of GR, the differences were not statistically significant. A possible explanation is that low doses of synthetic GCs, such as DEX and MP, poorly penetrate the blood-brain barrier due to the existence of P-glycoprotein, whereas CORT can freely cross the blood-brain barrier [41]. The GR and MR antagonists counteracted the opposite effects of MP and CORT, demonstrating receptor-mediated effects. Our results suggest that the balance of GR/MR activation is crucial for the survival of PVN neurons. MP and CORT exerted opposite effects on neurons in the PVN by activating different receptors.

The cell loss caused by secondary brain injury does not cease and lasts for weeks, years, or even the whole life after TBI [42]. Experimental studies have revealed that apoptosis contributed to approximately 30–60% of cell death after TBI [43, 44]. Most molecular mechanisms of secondary injury involve in the initiation of apoptosis by caspase-dependent or caspase-independent pathways, including hypoxia-ischemia, excitotoxicity, calcium dysregulation, oxidative stress, inflammation, and trophic factor withdrawal [45]. Bcl-2 family proteins play indispensable roles in both caspase-dependent and caspase-independent apoptosis by regulating the permeability of the mitochondrial membrane. In addition, some intracellular signaling pathways also participate in regulating neuronal apoptosis after TBI [46]. Akt is a serine-threonine kinase that is widely expressed in the brain, including the hippocampus, and has been reported to be involved in the process of secondary injury after TBI [20, 21]. Increased phosphorylation of Akt plays a crucial role in promoting neuronal survival and inhibiting apoptosis. Both experimental and clinical studies have found that activated Akt exerted antiapoptotic effects by inactivating Bad, which could bind to bcl-2 and release bax. Crosstalk between these pathways is very common. For example, CREB can be phosphorylated on the Ser-133 residue by PI3K/Akt, cyclic AMP-dependent protein kinase/protein kinase-A, protein kinase-C, and MAPK/ERK. The transcription factor CREB has been reported to be activated by Akt and exerts neuroprotective effects by upregulating the expression of downstream target proteins, including BDNF and Bcl-2 [47]. Vice versa, BDNF can increase the level of P-Akt by activating TrkB receptors and subsequently promotes neuronal survival. Interestingly, many studies have reported crosstalk between GCs and the TrkB/Akt signaling pathways [48]. However, the results of these studies are inconsistent or even contradictory due to different stress models, types of GCs, and dosing schedules. For example, a transient increase in GCs exhibits neuroprotective effects by activating TrkB/Akt signaling [49], while chronic stress or prolonged exogenous GC administration suppresses hippocampal BDNF and CREB expression, leading to neuronal apoptosis and memory impairment [50]. The effects of the GR and MR on Akt/CREB/BDNF and Bcl-2 family proteins in the PVN after TBI have not been investigated.

In the present study, we found that both the expression and activation of proapoptotic proteins (Bax and Bad) in the PVN were significantly upregulated on postinjury day 7, which was the peak time of apoptosis. Moreover, the level of the antiapoptotic protein Bcl-2 was reduced. The activation of the GR by high-dose MP exhibited proapoptotic effects by increasing the Bax levels, reducing the Bcl-2 levels, and activating Bad, while low-dose CORT replacement increased the expression of Bcl-2, inactivated Bad, and reduced the Bax levels by activating the MR. Accordingly, we showed that the GR and MR exhibited opposite effects on the activity of Akt/CREB/BDNF pathway. Activation of GR significantly inhibited the activation of Akt/CREB and reduced the expression of BDNF and Bcl-2. In contrast, increased activation of MR by low-dose CORT increased the activation of Akt/CREB and the expression of BDNF and Bcl-2. Our results suggest that Akt/CREB/BDNF might be the major signaling pathway that regulates Bcl-2 family proteins and neuronal apoptosis in the PVN after TBI. The use of RU486 and SPIRO counteracted the proapoptotic effect of MP and the antiapoptotic effect of CORT, respectively, indicating that their effects in apoptosis were mediated by the GR and MR.

### Limitations of this study

Our study only showed that the changes in the Bcl-2 family proteins and the Akt/CREB/BDNF pathway were mediated by the GR and MR but did not directly examine whether the Bcl-2 family proteins were regulated by the Akt/CREB/BDNF pathway. In addition, we did not investigate whether the activation of CREB was regulated by PI3K/Akt alone or together with other signaling pathways. Finally, evidence has shown the sex dependence of the corticosteroid receptor signaling pathways. However, we used only male rats in this study. Therefore, in a future study, we will further investigate the relationships between the Akt and CREB pathways and the sex-specific effects of GR and MR.

## Conclusions

In conclusion, our results showed that GR and MR are abundantly coexpressed in the PVN. The balance of GR/MR after TBI is crucial for the survival of PVN neurons and plays an important role in maintaining normal function of the HPA axis. High-dose MP, which has been commonly used in TBI patients but has subsequently been proven to be harmful, exhibits GR-mediated deleterious effects on the survival of PVN cells, leading to an increased incidence of CIRCI and mortality after TBI. However, treatment with a low dose of the MR agonist CORT restored the reduced plasma levels of CORT and MR activation, protected PVN neurons, alleviated CIRCI, and reduced CIRCI-related mortality. Our study revealed the receptor-mediated mechanisms and the downstream signaling pathway of the opposite effects of different GCs, providing a theoretical basis for the rational application of MR agonists after TBI, especially for TBI patients with CIRCI.

## Data Availability

The datasets used and/or analyzed during the current study are available from the corresponding author on reasonable request.

## Abbreviations

BDNF: Brain-derived neurotrophic factor
CREB: cAMP-responsive element-binding protein
CCI: Controlled cortical impact
CII: CORT increase index
CRH: Corticotropin-releasing hormone
CIRCI: Critical illness-related corticosteroid insufficiency
DG: Dentate gyrus
DEX: Dexamethasone
ES: Electrical stimulation
GR: Glucocorticoid receptor
GCs: Glucocorticoids
HPA: Hypothalamus-pituitary-adrenal axis
MP: Methylprednisolone
MR: Mineralocorticoid receptor
PVN: Paraventricular nucleus
PKB (Akt): Protein kinase B
TBI: Traumatic brain injury
